# Prognostic Value of Cardiac Troponin I in Patients with Ventricular Tachyarrhythmias

**DOI:** 10.3390/jcm11112987

**Published:** 2022-05-25

**Authors:** Ibrahim Akin, Michael Behnes, Julian Müller, Jan Forner, Mohammad Abumayyaleh, Kambis Mashayekhi, Muharrem Akin, Thomas Bertsch, Kathrin Weidner, Jonas Rusnak, Dirk Große Meininghaus, Maximilian Kittel, Tobias Schupp

**Affiliations:** 1 First Department of Medicine, University Medical Centre Mannheim (UMM), Faculty of Medicine Mannheim, University of Heidelberg, 68167 Mannheim, Germany; ibrahim.akin@umm.de (I.A.); jan.forner@umm.de (J.F.); mohammad.abumayyaleh@umm.de (M.A.); kathrin.weidner@umm.de (K.W.); jonas.rusnak@umm.de (J.R.); tobias.schupp@umm.de (T.S.); 2 Clinic for Interventional Electrophysiology, Heart Centre Bad Neustadt, 97616 Bad Neustadt a. d. Saale, Germany; julianmueller240491@gmail.com; 3 Department of Cardiology and Angiology, Philipps-University Marburg, 35037 Marburg, Germany; 4 Department of Internal Medicine and Cardiology, Mediclin Heart Centre Lahr, 77933 Lahr, Germany; kambis.mashayekhi@universitaets-herzzentrum.de; 5 Department of Cardiology and Angiology, Hannover Medical School, 30625 Hannover, Germany; akin-muharrem@mh-hannover.de; 6 Institute of Clinical Chemistry, Laboratory Medicine and Transfusion Medicine, Nuremberg General Hospital, Paracelsus Medical University, 90419 Nuremberg, Germany; thomas.bertsch@nuernberg.de; 7 Department of Cardiology, Carl-Thiem-Klinikum Cottbus, 03048 Cottbus, Germany; med1@ck.de; 8 Institute for Clinical Chemistry, Faculty of Medicine Mannheim, Heidelberg University, 68167 Mannheim, Germany; maximilian.kittek@umm.de

**Keywords:** ventricular tachyarrhythmias, cardiac troponin I, biomarkers, sudden cardiac death, coronary artery disease

## Abstract

Besides the diagnostic role in acute myocardial infarction, cardiac troponin I levels (cTNI) may be increased in various other clinical conditions, including heart failure, valvular heart disease and sepsis. However, limited data are available regarding the prognostic role of cTNI in the setting of ventricular tachyarrhythmias. Therefore, the present study sought to assess the prognostic impact of cTNI in patients with ventricular tachyarrhythmias (i.e., ventricular tachycardia (VT) and fibrillation (VF)) on admission. A large retrospective registry was used, including all consecutive patients presenting with ventricular tachyarrhythmias from 2002 to 2015. The prognostic impact of elevated cTNI levels was investigated for 30-day all-cause mortality (i.e., primary endpoint) using Kaplan–Meier, receiver operating characteristic (ROC), multivariable Cox regression analyses and propensity score matching. From a total of 1104 patients with ventricular tachyarrhythmias and available cTNI levels on admission, 46% were admitted with VT and 54% with VF. At 30 days, high cTNI was associated with the primary endpoint (40% vs. 22%; log rank *p* = 0.001; HR = 2.004; 95% CI 1.603–2.505; *p* = 0.001), which was still evident after multivariable adjustment and propensity score matching (30% vs. 18%; log rank *p* = 0.003; HR = 1.729; 95% CI 1.184–2.525; *p* = 0.005). Significant discrimination of the primary endpoint was especially evident in VT patients (area under the curve (AUC) 0.734; 95% CI 0.645–0.823; *p* = 0.001). In contrast, secondary endpoints, including all-cause mortality at 30 months and a composite arrhythmic endpoint, were not affected by cTNI levels. The risk of cardiac rehospitalization was lower in patients with high cTNI, which was no longer observed after propensity score matching. In conclusion, high cTNI levels were associated with increased risk of all-cause mortality at 30 days in patients presenting with ventricular tachyarrhythmias.

## 1. Introduction

Despite improvements in the treatment strategies of cardiovascular diseases, including better guideline adherence to pharmacological therapies, coronary revascularization strategies and increasing supply with an implantable cardioverter defibrillator (ICD), sudden cardiac death (SCD) still accounts for almost 50% of all cardiovascular deaths [1,2,3]. While congenital heart defects account for most SCD cases in younger patients, coronary artery disease (CAD) is the main cause for SCD-related deaths in patients over 35 years of age [4]. In more than 75% of cases, SCD occurs due to ventricular tachyarrhythmias (i.e., ventricular tachycardia (VT) or fibrillation (VF)). Commonly, VF is detected in patients with acute myocardial injury, leading to metabolic derangement and oxidative stress, whereas VT may occur more often in patients with structural heart disease (such as ischemic cardiomyopathy (ICMP)) and channelopathies [5]. However, in more than half of the patients, SCD occurs in patients without evidence of severe heart failure (HF). The mechanisms causing SCD are not fully understood, as outlined within recent European guidelines for the prevention of ventricular tachyarrhythmias and SCD [6,7]. Therefore, the identification of patients at high risk for SCD remains challenging [7]. Over the years, the identification of biomarkers for the identification of individuals at high risk for SCD (such as cardiac troponins, N-terminal pro-B-type natriuretic peptide (NT pro-BNP), Galectin-3 and soluble ST2) has gained greater significance [8,9,10,11,12].

Cardiac muscle contraction occurs as a result of increased intracellular Ca2+ levels, which affects the troponin complex—consisting of troponin C (cTNC), troponin T (cTNT) and troponin I (cTNI). The regulatory role of TNI consists in the inhibition of the Adenosine 5’-TriPhosphatase (ATPase) activity of the actomyosin complex and the modulation of cross-bridge formation and cardiac muscle contraction [13,14]. In particular, cTNT and cTNI are biomarkers of myocardial injury and are commonly released during myocardial necrosis in the presence of acute myocardial infarction (AMI) [15]. Myocardial necrosis causes the replacement of cardiac myocytes by fibrotic tissue, which further promotes adverse cardiac remodeling [16]. Elevated cTNI has already been shown to increase the risk of the composite endpoint of death, percutaneous coronary intervention (PCI) and AMI—especially in patients with a history of CAD—as observed in a study of 131 patients with supraventricular tachyarrhythmias [17]. In line with this finding, it was demonstrated that cTNT can function as a predictor of cardiac death, as was found among 70 patients with heart failure and left ventricular ejection fraction (LVEF) ≤ 35% at 2.2 years [18].

However, to the best of the authors’ knowledge, no available study has investigated the prognostic role of cardiac troponins in patients admitted with ventricular tachyarrhythmias. Therefore, this study investigates the prognostic role of cTNI levels on 30-day all-cause mortality (primary endpoint) in patients presenting with index ventricular tachyarrhythmias. Secondary endpoints include a composite arrhythmic endpoint (i.e., recurrent ventricular tachyarrhythmias, appropriate ICD therapies and SCD) and cardiac rehospitalization at 30 months.

## 2. Materials and Methods

### 2.1. Data Collection and Documentation

The present study retrospectively included data from all consecutive patients presenting with ventricular tachyarrhythmias on hospital admission, from 2002 to 2015, at the First Department of Medicine, University Medical Centre Mannheim, Germany, as recently published [19]. Using the hospital information system, all relevant clinical data related to the index event were documented.

Ventricular tachyarrhythmias comprised VT and VF, as defined by current international guidelines [7]. Sustained VT was defined by a duration of >30 s or as causing hemodynamic collapse within 30 seconds. Nonsustained VT was defined by a duration of <30 s. Both were characterized by wide QRS complexes (≥120 ms) at a rate greater than 100 beats per minute [7]. Ventricular tachyarrhythmias were documented by 12-lead electrocardiogram (ECG), ECG telemonitoring, ICD or—in the case of an unstable course or during cardiopulmonary resuscitation (CPR)—by external defibrillator monitoring. Documented VF was treated by external defibrillation and—in case of prolonged instability— with additional intravenous antiarrhythmic drugs during CPR.

The present study is derived from an analysis of the “Registry of Malignant Arrhythmias and Sudden Cardiac Death-Influence of Diagnostics and Interventions (RACE-IT)” and represents a single-center registry, including consecutive patients presenting with ventricular tachyarrhythmias and aborted cardiac arrest being acutely admitted to the University Medical Center Mannheim (UMM), Germany (clinicaltrials.gov; identifier: NCT02982473), from 2002 to 2015. The registry was carried out according to the principles of the declaration of Helsinki and was approved by the medical ethics committee II of the Medical Faculty Mannheim (Ethical Approval Number: 2016-612N-MA), University of Heidelberg, Germany.

### 2.2. Measurement of cTNI

During the study period from 2002 to 2015, cTNI testing was performed using three different cTNI assays. From 2002 to June 2006, the SIEMENS Dimension RxL CTNI assay was used for cTNI testing. The lowest detection limit of the assay was 0.004 ng/mL. The 99th percentile, measured from a healthy reference population, was 0.007 ng/mL, with a coefficient of variation (CV) of 15–22%. Thereafter, from June 2006 to December 2010, the Beckman Coulter Access AccuTNI assay was used, with the lowest detection limit being 0.01 ng/mL. The 99th percentile, measured from a healthy reference population, was 0.04 ng/mL, with a CV of 10%. From December 2010 to the end of the study period, cTNI was measured with the SIEMENS Dimension^®^ Vista 1500™. The lowest detection limit of the assay was 0.015 ng/mL. The 99th percentile, measured from a healthy reference population, was 0.045 ng/mL, with a CV of 10% [20].

### 2.3. Definition of Study Groups, Inclusion and Exclusion Criteria

For the present analysis, risk stratification was performed according to a single cTNI measurement related to the index event. For each patient, only the cTNI measurement closest to the index event was used, with a maximum time frame of 24 h before and after the index event. First, risk stratification was performed, dichotomized according to the median cTNI level. Despite the use of three different cTNI assays during the study period, the median cTNI level for each cTNI assay was assessed. Patients were classified as “high cTNI” (in the presence of cTNI above the median cTNI level) and “low cTNI” (in the presence of cTNI below or equal the median cTNI level) for each of the cTNI assays. To better assess the prognostic value of incremental cTNI increase, quartile analyses were performed thereafter. Quartiles were calculated, separated by each cTNI assay. Accordingly, patients were classified as “low” (Q1), “low-intermediate” (Q2), “intermediate-high” (Q3) and “high” (Q4). Based on those quartiles, the prognostic impact of incremental cTNI increase was investigated within the entire study cohort, and thereafter within prespecified subgroups. Patients without cTNI measurement during the allowed time frame, as well as patients without complete follow-up data regarding mortality, were excluded. Each patient was included only once when presenting with the first episode of ventricular tachyarrhythmias.

### 2.4. Risk Stratification

Further risk stratification was performed according to the underlying cardiac pathology. Patients with non-AMI, ST-segment elevation myocardial infarction (STEMI), non-ST segment elevation myocardial infarction (NSTEMI), ischemic (ICMP) and nonischemic cardiomyopathy (NICMP), as well as patients with idiopathic ventricular tachyarrhythmias, were analyzed.

STEMI was defined as a novel rise in the ST segment in at least two contiguous leads, with ST-segment elevation ≥ 2.5 mm in men < 40 years, ≥ 2 mm in men ≥ 40 years, or ≥ 1.5 mm in women in leads V2–V3 and/or 1 mm in the other leads. Additional ECG criteria were new ST depression or inversion, T wave alterations, Q waves or new left bundle branch block [21]. NSTEMI was defined as the presence of an acute coronary syndrome with a troponin I increase of above the 99th percentile of a healthy reference population, in the absence of ST segment elevation, but with persistent or transient ST segment depression, inversion or alteration of T wave, or a normal ECG in the presence of a coronary culprit lesion. The culprit lesion was defined as an acute complete thrombotic occlusion for STEMI and as any relevant critical coronary stenosis for NSTEMI, with the potential need for coronary revascularization either by PCI or coronary artery bypass grafting (CABG). The presence of a coronary culprit lesion was mandatory for both diagnoses of NSTEMI and STEMI. Evidence of regional wall motion abnormalities was also included in AMI diagnosis, as far as was available. Values of left ventricular ejection fraction (LVEF) were retrieved from standardized transthoracic echocardiographic examinations, usually performed before hospital discharge in survivors, to assess realistic LVEF values beyond the acute phase of acute coronary ischemia during AMI. In minor part, and only if available, earlier LVEF values, assessed on admission or during intensive care, were retrieved from patients who died while already within the acute phase of AMI [22].

ICMP comprised all patients with LVEF < 55% and had either prior documented CAD or newly diagnosed CAD, as well as patients with AMI assessed by coronary angiography at index stay sufficient to cause myocardial dysfunction. Identification of CAD (defined as at least one relevant stenosis of one epicardial coronary artery of more than 50%) was based on the judgment of the investigating interventional cardiologist during routine care. All coronary angiograms and reports were reassessed post hoc by two independent interventional cardiologists to determine whether the CAD was sufficient for causality of myocardial dysfunction [23]. NICMP comprised all patients with LVEF < 55%, in the absence of CAD, valvular heart disease and congenital heart disease sufficient to cause the observed myocardial abnormality. The following types were allocated to the NICMP group: dilated cardiomyopathy (DCM), hypertrophic obstructive cardiomyopathy, arrhythmogenic right ventricular dysplasia (ARVD) and noncompaction cardiomyopathy (NCCMP) [23,24,25,26].

Patients presenting without AMI, ICMP and NICMP, and who had no evidence of impaired LVEF or structural heart disease, were classified as patients with “idiopathic ventricular tachyarrhythmias”.

Finally, the prognostic impact of cTNI was investigated within different subgroups of patients with CAD, whereas only patients undergoing coronary angiography were included. Thus, multivessel disease (MVD) was characterized by significant stenosis of at least 2 major coronary vessels (defined as at least one relevant stenosis of one epicardial coronary artery of more than 50% and/or prior PCI of one coronary artery). The coronary chronic total occlusion (CTO) group comprised all patients with a native unrevascularized CTO in coronary vessels with a diameter >1.5 mm [27,28]. Identification of CTO and CAD was based on the judgment of the investigating interventional cardiologist during routine care. The CTO group also included patients with acute revascularization of non-CTO-vessels by PCI or CABG. Moreover, patients with an occluded bypass graft on the native CTO vessel were allocated to CTO group.

### 2.5. Study Endpoints

The primary endpoint was all-cause mortality at 30 days after index ventricular tachyarrhythmias. Secondary endpoints were all-cause deaths at 24 h, 30 months, cardiac death at 30 days, cardiac rehospitalization at 30 months and a composite arrhythmic endpoint (including recurrent ventricular tachyarrhythmias, appropriate ICD therapies and SCD) at 30 days and 30 months after index ventricular tachyarrhythmias. Overall follow-up period lasted until 2016. All-cause mortality was documented using our electronic hospital information system and by directly contacting state resident registration offices (“bureau of mortality statistics”) across Germany. Identification of patients was verified by place of name, surname, day of birth and registered living address. Lost-to-follow-up rate was 1.7% (*n* = 48) regarding survival until the end of the follow-up period.

### 2.6. Statistical Methods

Quantitative data are presented as mean ± standard error of mean (SEM), median and interquartile ranges (IQR), as well as ranges depending on the distribution of the data and were compared using the Student’s t test for normally distributed data, or the Mann–Whitney U test for nonparametric data. Deviations from a Gaussian distribution were tested by the Kolmogorov–Smirnov test. Spearman’s rank correlation for nonparametric data was used to test univariate correlations. Qualitative data are presented as absolute and relative frequencies and were compared using the Chi^2^ test or the Fisher’s exact test, as appropriate.

Firstly, overall data of consecutive patients on admission are given for the entire unmatched cohort in order to present the real-life character of health-care supply at our institution between 2002 and 2015. Here, Kaplan–Meier method, as well as uni- and multivariable Cox regression models, were applied for the evaluation of all-cause mortality at 30 days after index ventricular tachyarrhythmias.

Secondly, propensity score matching was applied. Propensity scores (1:1) were created for the comparisons of “high cTNI” vs. “low TNI”, including the entire study cohort and applying a nonparsimonious multivariable logistic regression model. Propensity scores were created according to the presence of the following independent variables: age, sex, diabetes, chronic kidney disease, CAD, LVEF, CPR, index ventricular tachyarrhythmia (i.e., VT/VF) and presence of an ICD. Based on the propensity score values counted by logistic regression, for each patient, one patient in the control group with a similar propensity score value was found (accepted difference of propensity score value: <5%). Univariable stratification was performed using the Kaplan–Meier method, with comparisons between groups using univariable hazard ratios (HR) given together with 95% confidence intervals.

Finally, the prognostic value of cTNI, assessed with the SIEMENS Dimension^®^ Vista intelligent lab system, was investigated using receiver operating characteristic (ROC) analyses. ROC analyses were performed separately for both patients with index episodes of VT and VF. An optimum cutoff value was determined in accordance with the maximum Youden index. The Youden index, defined as the maximum of sensitivity + specificity –1, was used to determine the largest total diagnostic accuracy a biomarker can achieve [29,30].

The result of a statistical test was considered significant for *p* < 0.05. SPSS (Version 25, IBM Armonk, New York, NY, USA) was used for statistics.

## 3. Results

### 3.1. Entire Study Cohort

**Figure 1:**
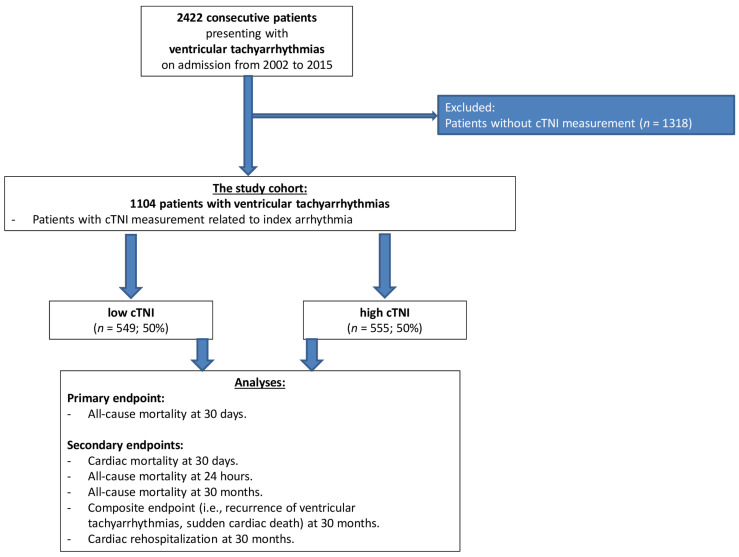
Study population. cTNI, cardiac troponin I.

From 2422 patients with ventricular tachyarrhythmias on admission, 1318 patients without cTNI measurement related to the index event were excluded. Accordingly, the present study included 1104 patients with ventricular tachyarrhythmias and available cTNI measurement (Figure 1).

**Figure 2:**
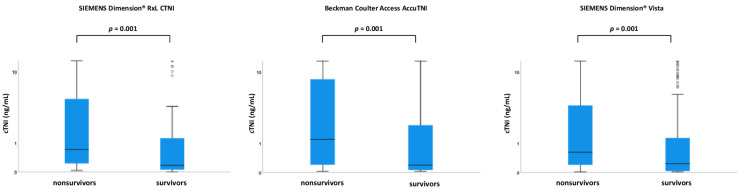
Box plots demonstrating distribution of cTNI levels depending on the applied assay, in patients with ventricular tachyarrhythmias, comparing survivors and nonsurvivors at 30 days.

Within the entire study cohort, median cTNI levels were 0.700 ng/mL (IQR 0.370–3.125 ng/mL) for the SIEMENS Dimension^®^ RxL CTNI assay, 0.800 ng/mL (IQR 0.300–3.515 ng/mL) for the Beckman Coulter Access AccuTNI assay and 0.610 ng/mL (IQR 0.380–2.2965 ng/mL for the SIEMENS Dimension^®^ Vista 1500™ assay. Accordingly, cTNI did not significantly differ among the different cTNI assays (*p* ≥ 0.248 for all comparisons). As illustrated in Figure 2, cTNI levels were significantly higher among nonsurvivors as compared to survivors at 30 days, irrespective of the applied cTNI assay (*p* = 0.001 for all comparisons).

Patients’ characteristics within the entire, unmatched study cohort for the comparison of patients with high vs. low cTNI are outlined within Table 1 (left panel). Patients with high cTNI more frequently presented with VF as compared to patients with low cTNI (62% vs. 47%; *p* = 0.001). In contrast, cardiovascular risk factors and LVEF were equally distributed in both groups. Rates of CPR were higher in patents with elevated cTNI (70% vs. 49%; *p* = 0.001). Furthermore, patients with high cTNI were more frequently treated with beta blockers, ACE inhibitors, statins, amiodarone and aldosterone antagonists.

CAD-related findings are presented in Table 2 (left panel). Coronary angiography was more frequently performed in patients with high cTNI (75% vs. 63%; *p* = 0.001), who had higher rates of CAD (87% vs. 67%; *p* = 0.001). In line with this finding, the rate of PCI was significantly higher among patients with elevated cTNI (72% vs. 37%; *p* = 0.001).

### 3.2. Survival Analyses within the Entire Study Cohort

**Figure 3:**
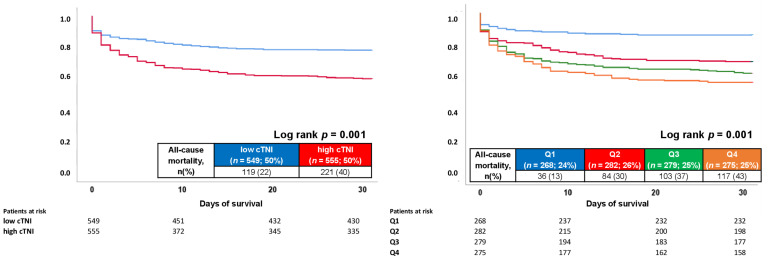
Kaplan–Meier analysis comparing patients with high cTNI to patients with low cTNI (**left** panel), as well as within a quartile analysis (**right** panel), with regard to 30-day all-cause mortality (primary endpoint) within the entire unmatched study cohort.

Median follow-up time in the entire study cohort was 1.7 years (IQR 8 days–5.0 years). At 30 days, the all-cause mortality (primary endpoint) occurred in 40% of patients with high cTNI and in 20% of patients presenting with low cTNI (Figure 3).

Accordingly, elevated cTNI levels were associated with increased 30-day all-cause mortality (primary endpoint) in patients with ventricular tachyarrhythmias (HR = 2.004; 95% CI 1.603–2.505; *p* = 0.001) (Table 3, left). Furthermore, cardiac death was more common in patients with high cTNI (31% vs. 17%; *p* = 0.001). In line with this finding, higher rates of all-cause mortality at 24 hours (22% vs. 13%; *p* = 0.001) and 30 months (52% vs. 38%; *p* = 0.001) were seen in patients with high cTNI. The risk of the composite arrhythmic endpoint (i.e., recurrent ventricular tachyarrhythmias, appropriate ICD therapies and SCD) and cardiac rehospitalization at 30 months were not affected by cTNI within the unmatched study cohort. Notably, median intensive care unit (ICU) time was longer in patients with high cTNI (4 days (interquartile range (IQR) 2–10 days) vs. 4 days (IQR 1–8 days); *p* = 0.001), along with a shorter median follow-up time (624 vs. 1079 days) (Table 3, left panel).

**Figure 4:**
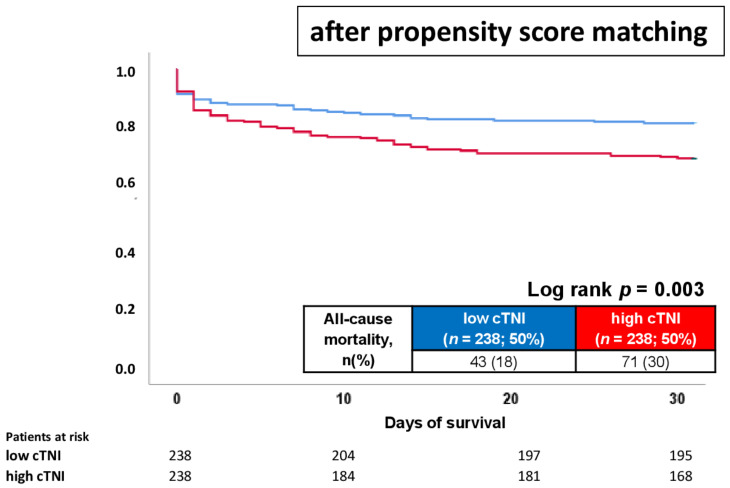
Kaplan–Meier analysis comparing patients with high cTNI to patients with low cTNI with regard to 30-day all-cause mortality (primary endpoint) within the propensity-matched cohort.

Despite significant differences regarding the distribution of index tachyarrhythmias and comorbidities among patients with and without elevated cTNI levels, additional propensity score-matched analyses were performed (*n* = 238 patients with high and low cTNI). After propensity score matching (Table 1 and Table 2, right panels), no further differences were observed regarding age, distribution of VT/VF and cardiovascular risk factors. Especially for LVEF, distribution of CAD and ICD rates were comparable in both groups. After propensity score matching, cTNI was still associated with increased risk of 30-day all-cause mortality (30% vs. 18%; log rank *p* = 0.003; HR = 1.729; 95% CI 1.184–2.525; *p* = 0.005) (Table 3; right panel; Figure 4). However, rates of all-cause mortality at 24 hours (16% vs. 11%; *p* = 0.179) and 30 months (42% vs. 36%; *p* = 0.133), as well as risk of the composite arrhythmic endpoint (19% vs. 15%; *p* = 0.272) and cardiac rehospitalization (8% vs. 13%; *p* = 0.073), did not differ in patients with and without elevated cTNI levels following propensity score matching (Table 3; right panel). Furthermore, follow-up times were significantly shorter (median 1006 vs. 1258 days; *p* = 0.001) in patients with high cTNI, whereas hospitalization and ICU times did not differ among patients with high or low cTNI (Table 3; right panel).

Subsequently, quartile analyses were performed within the entire, unmatched study cohort, whereas the following groups were analyzed: Q1: SIEMENS Dimension^®^ RxL CTNI assay < 0.370 ng/mL; Beckman Coulter Access AccuTNI < 0.300 ng/mL; SIEMENS Dimension^®^ Vista 1500™ < 0.380 ng/mL; Q2: SIEMENS Dimension^®^ RxL CTNI assay 0.370–0.700 ng/mL; Beckman Coulter Access AccuTNI 0.300–0.800 ng/mL; SIEMENS Dimension^®^ Vista 1500™ 0.380–610 ng/mL; Q3: SIEMENS Dimen-sion^®^ RxL CTNI assay > 0.700–3.125 ng/mL; Beckman Coulter Access AccuTNI > 0.800–3.515 ng/mL; SIEMENS Dimension^®^ Vista 1500™ > 0.610–2.2965 ng/mL; Q4: SIEMENS Dimension^®^ RxL CTNI assay >3.125 ng/mL; Beckman Coulter Access AccuTNI > 3.515 ng/mL; SIEMENS Dimension^®^ Vista 1500™ > 2.2965 ng/mL.

When analyzed as quartile analyses within the entire, unmatched study cohort, patients with high cTNI were associated with highest all-cause mortality at 30 days (HR = 3.639; 95% CI 2.504–5.288; *p* = 0.001), followed by patients with intermediate-high cTNI (HR = 3.071; 95% CI 2.101–4.489; *p* = 0.001) and low-intermediate cTNI (HR = 2.387; 95% CI 1.615–3.527; *p* = 0.001), as compared to patients with low cTNI (Figure 3; right panel).

### 3.3. Survival Analyses within Prespecified Subgroups

**Figure 5:**
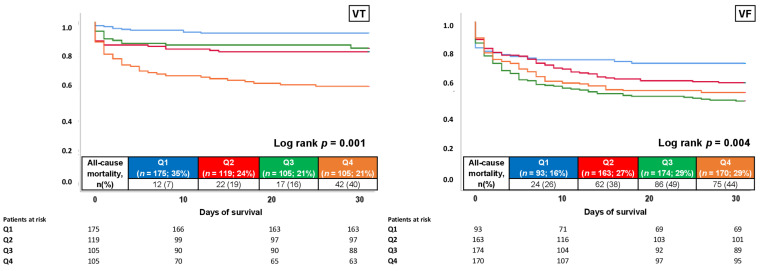
Kaplan–Meier analysis for cTNI with regard to the 30-day all-cause mortality (primary endpoint), stratified by patients with VT (**left** panel) and VF (**right** panel).

When focusing on prespecified subgroups within the unmatched study cohort, increased risk of mortality at 30 days in patients with high cTNI was observed in both patients admitted with index episodes of VT (HR = 2.694; 95% CI 1.762–4.121; *p* = 0.001) and VF (HR = 1.496; 95% CI 1.151–1.944; *p* = 0.004) (Figure 5).

**Figure 6:**
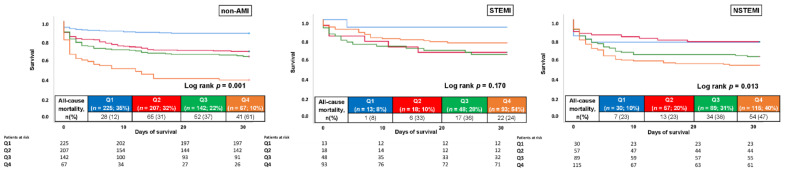
Kaplan–Meier analysis for cTNI with regard to 30-day all-cause mortality (primary endpoint), stratified by the presence or absence of AMI.

When stratified by the presence of AMI, no prognostic impact of cTNI was observed in patients with STEMI (HR = 1.258; 95% CI 0.563–2.813; *p* = 0.576), whereas patients with NSTEMI (HR = 2.030; 95% CI 1.249–3.300; *p* = 0.004) and non-AMI (HR = 2.364; 95% CI 1.772–3.152; *p* = 0.001) had increased risk of 30-day all-cause mortality when presenting with elevated cTNI (Figure 6).

**Figure 7:**
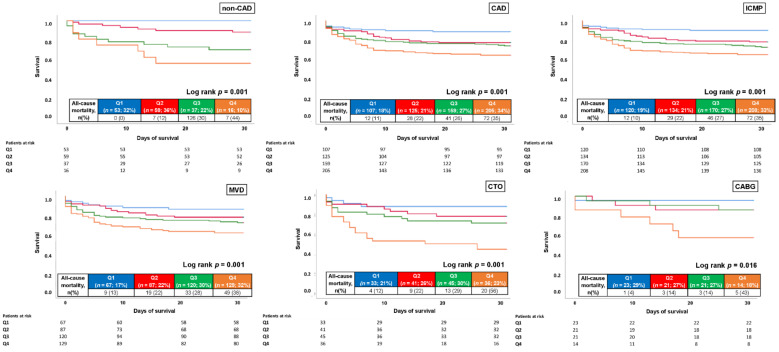
Kaplan–Meier analysis for cTNI with regard to 30-day all-cause mortality (primary endpoint) in different subgroups undergoing coronary angiography.

Subsequently, prognosis of cTNI was investigated within the subgroup of patients undergoing coronary angiography during index hospitalization. In patients undergoing coronary angiography, high cTNI was associated with increased risk of 30-day mortality in patients with no CAD (HR = 6.421; 95% CI 2.679–15.390; *p* = 0.001) and CAD (HR = 1.940; 95% CI 1.353–2.783; *p* = 0.001), and especially in those with MVD (HR = 1.985; 95% CI 1.292–3.048; *p* = 0.002), CTO (HR = 2.638; 95% CI 1.388–5.016; *p* = 0.003) and ICMP (HR = 2.106; 95% CI 1.476–3.004; *p* = 0.001) (Figure 7).

Finally, high cTNI was not significantly associated with 30-day all-cause mortality in patients with NICMP (HR = 6.299; 95% CI 0.703–56.414; *p* = 0.100), whereas all-cause mortality was increased in patients with idiopathic VT/VF in the presence of elevated cTNI (HR = 2.674; 95% CI 1.730–4.135; *p* = 0.001) (Supplemental Figure S1).

### 3.4. Multivariable Cox Regression Analysis

Even after multivariable adjustment within the entire, unmatched study cohort, high cTNI was associated with increased risk of all-cause mortality at 30 days (HR = 1.541; 95% CI 1.088–2.182; *p* = 0.0015). Besides cTNI, increased age (HR = 1.141; *p* = 0.042), chronic kidney disease (HR = 5.786; *p* = 0.001) and LVEF < 35% (HR = 1.260; *p* = 0.002) were particularly associated with increased risk of death, whereas the presence of an ICD (HR = 0.096; *p* = 0.001), electrophysiological examination (HR = 0.261; *p* = 0.001) and coronary angiography (HR = 0.505; *p* = 0.001) were associated with favorable outcomes at 30 days (Table 4).

Even when analyzed within important subgroups, both patients with index episodes of VT (HR = 2.333; 95% CI 1.276–4.276; *p* = 0.006) and VF (HR = 1.708; 95% CI 1.177–2.478; *p* = 0.005) had increased all-cause mortality at 30 days in the presence of increased cTNI (Table 5). This was still demonstrated in patients with idiopathic ventricular tachyarrhythmias (HR = 2.628; 95% CI 1.223–5.651; *p* = 0.013) and NICMP (HR = 12.164; 95% CI 0.999–148.093; *p* = 0.050).

Furthermore, cTNI was associated with adverse outcomes in patients without AMI (HR = 1.963; 95% CI 1.331–2.897; *p* = 0.001) and NSTMI (HR = 2.661; 95% CI 1.126–6.289; *p* = 0.026), whereas cTNI had no prognostic impact in patients with STEMI (HR = 5.047; 95% CI 0.657–38.607; *p* = 0.120). Finally, patients with ischemic cardiomyopathy (HR = 1.801; 95% CI 1.102–2.942; *p* = 0.019) had especially increased risk of 30-day all-cause mortality when presenting with increased cTNI (Table 5).

### 3.5. Receiver Operating Characteristic (ROC) Analyses

**Figure 8:**
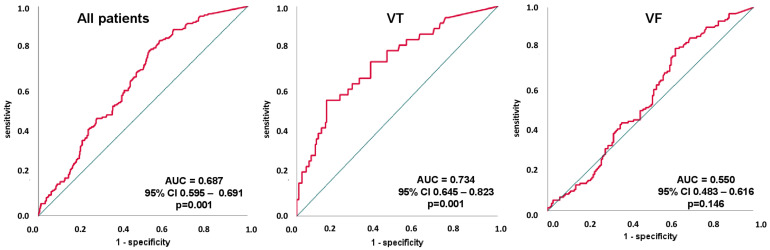
Receiver operator characteristic (ROC) curve analyses of cTNI for the prediction of 30-day all-cause mortality, stratified by patients within the entire study cohort, as well as separated by VT and VF.

The ROC analyses were performed within the entire, unmatched study cohort, as well as separately for patients with VT and VF. Despite the different cTNI assays, only cTNI values assessed by the SIEMENS Dimension^®^ Vista 1500™ cTNI assay were included within this analysis. Thus, cTNI showed a moderate predictive value for mortality at 30 days following ventricular tachyarrhythmias (area under the curve (AUC) 0.687; 95% CI 0.595–0.691; *p* = 0.001) within the entire study cohort. However, cTNI was a more reliable predictive value in patients admitted with VT (AUC 0.734; 95% CI 0.645–0.823; *p* = 0.001). A cTNI level of 2.3105 ng/mL was determined to be the best cutoff value, with a sensitivity of 55% and a specificity of 85%, respectively. In contrast, cTNI was not predictive in the presence of VF (AUC 0.550; 95% CI 0.483–0.616; *p* = 0.166) (Figure 8).

Finally, cTNI assessed by the SIEMENS Dimension^®^ Vista 1500™ cTNI assay showed reliable predictions of 30-day all-cause mortality in patients with coronary artery disease (AUC 0.684; 95% CI 0.598–0.769; *p* = 0.044) and NSTEMI (AUC 0.627; 95% CI 0.530–0.724; *p* = 0.050), whereas cTNI was not predictive for all-cause mortality in STEMI patients (AUC 0.559; 95% CI 0.429–0.690; *p* = 0.379) (Supplemental Table S1). Furthermore, cTNI showed good prediction of 30-day all-cause mortality in patients with idiopathic VT/VF (AUC 0.726; 95% CI 0.632–0.820; *p* = 0.001).

## 4. Discussion

The present study evaluated the prognostic impact of cTNI levels on 30-day all-cause mortality in patients presenting with ventricular tachyarrhythmias on hospital admission. This study suggests that there is increased short-term mortality in patients with increased cTNI, which was seen in patients admitted both with episodes of VT and VF. These findings were consistent, even after multivariable adjustment and propensity score matching. Patients with CAD, ICMP and MVD especially had increased risk of death in the presence of elevated cTNI. Even in VT patients, cTNI demonstrated valuable discrimination of the primary endpoint, with an AUC of 0.734. In contrast, long-term prognostic endpoints (including all-cause mortality and risk of the composite arrhythmic endpoint) were not affected by single cTNI measurements. Surprisingly, risk of cardiac rehospitalization was lower in patients with high cTNI, which was no longer observed after propensity score matching.

Although the identification of patients at high risk for SCD is of major clinical interest, data on the prognostic role of biomarkers in this setting are still limited [31]. Within a large case–control study that included six prospective cohorts and 565 SCD cases (as compared to 1090 matched controls), cTNI, NT-proBNP, high-density lipoprotein cholesterol ratio and high-sensitivity C-reactive protein were especially associated with an increased risk of SCD at 11 years [6]. In line with this finding, a serial increase from baseline cTNI was demonstrated to predict SCD in more than 3000 ambulatory patients who were included in the “Cardiovascular Health Study” [8].

In contrast, to the best of our knowledge, no currently available study has investigated the prognostic impact of cTNI in patients with ventricular tachyarrhythmias on hospital admission. However, some studies have already evaluated the prognostic value of cTNI in patients following CPR with heterogeneous findings. Within a single-center retrospective registry, including 277 patients admitted after out-of-hospital cardiac arrest (OHCA), peak troponin, but not initial troponin was especially associated with an increased risk of PCI. Both initial and peak troponin did not affect the risk of in-hospital death [32]. However, in that study, only 58% of the patients initially had a shockable rhythm, and no further subanalyses were performed with regard to patients with ventricular tachyarrhythmias. On the contrary, a targeted temperature management (TTM) trial substudy composed of 669 OHCA patients suggested that hs-TNT was associated with increased risk of cardiovascular death and multiorgan failure, while the proportion of patients with initial shockable rhythm was higher (79%) [33]. However, the present study has a different point of view, as it included patients with ventricular tachyarrhythmias and only 35% of the patients were admitted to the hospital following OHCA.

Within our study, elevated cTNI levels were associated with increased risk of short-term death, especially in patients with VT, and cTNI was revealed to be a reliable predictor of all-cause deaths. By now, it is well understood that VT occurs particularly in patients with adverse structural remodeling, mainly related to pre-existing CAD, leading to scar-mediated re-entry [34]. Especially due to advances in AMI treatment related to nationwide health-care supply, shorter door-to-balloon times and improved pharmacotherapies following AMI, the number of patients suffering from ischemic heart disease, as well as the number of patients with arrhythmic substrate, have increased [35]. This raises the relevance of improved therapies for VT (such as catheter ablation) and better identification of patients who are at high risk of death. Therefore, this study identifies the cTNI level as an independent predictor of all-cause mortality in patients with VT, with a potentially increased arrhythmic burden. Thus, cTNI was shown to be a good predictor for the need of PCI in patients with CAD, and was found to increase the risk of death in patients with CAD, especially in 3-vessel CAD.

Furthermore, several studies have investigated the prognostic role of cTNI with regard to the occurrence of ventricular tachyarrhythmias. For instance, Liu et al. found that elevated cTNI levels were associated with the occurrence of nonsustained VT in 755 patients with hypertrophic obstructive cardiomyopathy [36]. However, cTNI did not affect the risk of the composite arrhythmic endpoint (i.e., recurrent ventricular tachyarrhythmias, appropriate ICD therapies and SCD) at long-term follow-up. Further studies are necessary to prove the association of cTNI in ventricular tachyarrhythmias, especially focusing on sequential cTNI measurements.

This study has several limitations. Despite the retrospective study design, there may be some confounding due to unmeasured confounding variables, even though we adjusted for potential confounding factors using multivariable Cox regression and propensity score-matching analyses. Patients with ventricular tachyarrhythmias, who were not admitted to our institution due to nonresuscitated OHCA, were beyond the scope of the present study. Although we investigated the prognostic impact of cTNI in different CAD subgroups, important tools to assess the severity of CAD, such as the “Synergy between Percutaneous Coronary Intervention with Taxus and Cardiac Surgery” (SYNTAX) II score, were beyond the scope of the present study. For the present study, risk stratification was performed according to a single cTNI measurement. The cTNI levels during follow-up were only available for a minor portion of the study population, and were therefore beyond the scope of this study. No exact time from arrhythmia-to-cTNI measurement is available for the present study. In 90.6% of the patients, cTNI was assessed at the same day of the index event. Even after investigating the prognostic role of cTNI in patients who had their cTNI assessment on index day, the findings were consistent within both the unmatched and the matched cohorts. Furthermore, minor confounding may be present despite the use of different cTNI assays during the study period. Based on the different types of cTNI assays, ROC analyses were restricted to the SIEMENS Dimension^®^ Vista intelligent lab system for contemporary sensitive cTNI testing. The mode of death was not available in 16% of the patients. Finally, despite the single-center study design, cardiac rehospitalization was assessed only at our institution.

## 5. Conclusions

In conclusion, in patients admitted with ventricular tachyarrhythmias, cTNI was associated with increased risk of all-cause mortality at 30 days. Especially in patients presenting with index episodes of VT, cTNI was a reliable predictor of all-cause death at 30 days.

## Figures and Tables

**Table 1:** Baseline characteristics before and after propensity score matching.

	Without Propensity Score Matching					With Propensity Score Matching				
Characteristic	Low cTNI (*n* = 549; 50%)		High cTNI (*n* = 555; 50%)		*p* Value	Low cTNI (*n* = 238; 50%)		High cTNI (*n* = 238; 50%)		*p* Value
**Age**, median (range)	67 (16–94)		67 (15–97)		0.474	67 (19–94)		67 (15–91)		0.415
**Male gender**, *n* (%)	397	(72)	402	(72)	0.965	178	(75)	174	(73)	0.676
**Ventricular tachyarrhythmias at index**, *n* (%)										
Ventricular tachycardia	293	(53)	211	(38)	**0.001**	116	(49)	110	(46)	0.582
Ventricular fibrillation	256	(47)	344	(62)		122	(51)	128	(54)	
**Underlying cardiac disease**, *n* (%)										
Coronary artery disease	235	(43)	108	(19)	**0.001**	91	(39)	84	(35)	0.436
STEMI	31	(6)	141	(25)	**0.001**	24	(10)	43	(18)	**0.012**
NSTEMI	87	(16)	204	(37)	**0.001**	61	(26)	47	(20)	0.125
Nonischemic cardiomyopathy	24	(4)	16	(3)	0.186	15	(6)	16	(7)	0.853
Channelopathy	23	(4)	10	(2)	**0.020**	9	(4)	8	(3)	0.805
Idiopathic ventricular tachyarrhythmias	149	(27)	76	(24)	**0.001**	38	(16)	40	(17)	0.804
**Cardiovascular risk factors**, *n* (%)										
Arterial hypertension	311	(57)	320	(58)	0.735	137	(58)	139	(58)	0.853
Diabetes mellitus	124	(23)	151	(27)	0.076	60	(25)	63	(27)	0.753
Hyperlipidemia	143	(26)	138	(25)	0.652	63	(27)	71	(30)	0.415
Smoking	161	(29)	188	(34)	0.104	77	(32)	84	(35)	0.498
Cardiac family history	48	(9)	52	(9)	0.717	22	(9)	19	(12)	0.300
**Comorbidities at index stay**, *n* (%)										
Prior myocardial infarction	129	(24)	109	(20)	0.119	53	(22)	57	(24)	0.664
Prior coronary artery disease	224	(41)	197	(36)	0.070	101	(42)	103	(43)	0.853
Prior heart failure	126	(23)	91	(16)	**0.001**	65	(27)	57	(24)	0.401
Prior PCI	121	(22)	98	(18)	0.068	60	(25)	52	(22)	0.387
Atrial fibrillation	170	(31)	170	(31)	0.904	76	(32)	88	(37)	0.247
Cardiopulmonary resuscitation	271	(49)	390	(70)	**0.001**	130	(55)	144	(61)	0.377
In hospital	97	(18)	142	(36)		49	(21)	48	(20)	
Out of hospital	174	(32)	248	(47)		81	(35)	96	(40)	
Chronic kidney disease	296	(54)	384	(69)	**0.001**	148	(62)	157	(66)	0.390
COPD	53	(10)	50	(9)	0.713	19	(8)	23	(10)	0.518
**LVEF**, *n* (%)										
>55%	142	(34)	114	(28)	0.073	71	(30)	69	(29)	0.531
54–45%	49	(12)	68	(17)		29	(12)	37	(16)	
44–35%	78	(19)	86	(21)		47	(20)	53	(22)	
<35%	147	(35)	133	(33)		91	(38)	79	(33)	
No evidence of LVEF		-		-	-	-	-	-	-	-
**Cardiac therapies at index**, *n* (%)										
Electrophysiological examination	100	(18)	47	(5)	**0.001**	34	(14)	16	(7)	**0.007**
VT ablation therapy	21	(4)	11	(2)	0.068	11	(5)	7	(3)	0.336
**Presence of an ICD at discharge**, *n* (%)	210	(50)	121	(37)	**0.001**	86	(46)	78	(48)	0.659
**Medication at discharge**, *n* (%)										
Beta blocker	332	(79)	291	(90)	**0.001**	164	(87)	144	(88)	0.657
ACE inhibitor	257	(61)	237	(73)	**0.001**	122	(65)	113	(69)	0.343
ARB	58	(14)	23	(7)	**0.003**	25	(13)	13	(8)	0.110
Statin	259	(62)	257	(79)	**0.001**	129	(68)	117	(72)	0.472
Amiodarone	51	(12)	56	(17)	**0.046**	24	(13)	34	(21)	**0.040**
Digitalis	41	(10)	20	(6)	0.078	18	(10)	15	(9)	0.918
Aldosterone antagonist	54	(13)	38	(12)	0.652	25	(13)	26	(16)	0.469

ACE, angiotensin converting enzyme; ARB, angiotensin receptor blocker; CAD, coronary artery disease; COPD, chronic obstructive pulmonary disease; cTNI, cardiac troponin I; left ventricular ejection fraction; NSTEMI, non-ST-segment elevation myocardial infarction; PCI, percutaneous coronary intervention; SEM, standard error of mean; VT, ventricular tachycardia. Bold type indicates *p* < 0.05.

**Table 2:** CAD-related findings.

	Without Propensity Score Matching					With Propensity Score Matching				
Characteristic	Low cTNI (*n* = 549; 50%)		High cTNI (*n* = 555; 50%)		*p* Value	Low cTNI (*n* = 238; 50%)		High cTNI (*n* = 238; 50%)		*p* Value
**Coronary angiography**, *n* (%)	344	(63)	417	(75)	**0.001**	161	(68)	180	(76)	0.053
No evidence of CAD	112	(33)	53	(13)	**0.001**	36	(22)	40	(22)	0.456
1-vessel disease	78	(23)	115	(28)		48	(30)	43	(24)	
2-vessel disease	84	(24)	139	(33)		43	(27)	61	(34)	
3-vessel disease	70	(20)	110	(26)		34	(21)	36	(20)	
**Significant stenosis of coronary vessels**, *n* (%)										
Right coronary artery	151	(44)	221	(53)	**0.012**	76	(47)	86	(48)	0.916
Left main trunk	17	(5)	32	(8)	0.126	9	(6)	12	(7)	0.680
Left anterior descending	156	(45)	246	(59)	**0.001**	89	(55)	91	(51)	0.383
Left circumflex	104	(30)	180	(43)	**0.001**	54	(34)	69	(39)	0.358
Chronic total occlusion	74	(22)	81	(19)	0.477	42	(26)	43	(24)	0.639
Presence of CABG	44	(13)	35	(8)	**0.048**	16	(10)	23	(13)	0.411
**PCI**, *n* (%)	127	(37)	300	(72)	**0.001**	76	(47)	89	(49)	0.680
Right coronary artery	57	(17)	106	(25)	**0.003**	31	(19)	36	(209	0.863
Left main trunk	6	(2)	20	(5)	**0.021**	3	(2)	3	(2)	1.000
Left anterior descending	64	(19)	153	(37)	**0.001**	41	(26)	47	(26)	0.892
Left circumflex	28	(8)	72	(17)	**0.001**	19	(12)	17	(9)	0.480
CABG	3	(0.9)	4	(1)	1.000	1	(0.6)	2	(1)	1.000
**Sent to CABG**, *n* (%)	10	(3)	7	(2)	0.254	5	(3)	5	(3)	1.000
**Thrombus aspiration**, *n* (%)	23	(7)	69	(17)	**0.001**	18	(11)	21	(12)	0.888
**CPR during coronary angiography**, *n* (%)	23	(7)	44	(11)	0.061	8	(5)	9	(5)	0.990

CABG, coronary artery bypass grafting; CAD, coronary artery disease; CPR, cardiopulmonary resuscitation; PCI, percutaneous coronary intervention. Bold type indicates *p* < 0.05.

**Table 3:** Endpoints and follow-up data before and after propensity score matching.

	Without Propensity Score Matching					With Propensity Score Matching				
Characteristics	Low cTNI (*n* = 549; 50%)		High cTNI (*n* = 555; 50%)		*p* Value	Low cTNI (*n* = 238; 50%)		High cTNI (*n* = 238; 50%)		*p* Value
**Primary endpoint**, *n* (%)										
All-cause mortality, at 30 days	119	(22)	221	(40)	**0.001**	43	(18)	71	(30)	**0.003**
**Secondary endpoints**, *n* (%)										
All-cause mortality, at 24 h	73	(13)	121	(22)	**0.001**	27	(11)	37	(16)	0.179
Cardiac death, at 30 days *	91/108	(17)	172/198	(31)	**0.001**	35/40	(15)	51/61	(21)	0.057
All-cause mortality, at 30 months	206	(38)	287	(52)	**0.001**	85	(36)	101	(42)	0.133
Cardiac rehospitalization, at 30 months	67	(12)	40	(7)	**0.005**	31	(13)	19	(8)	0.073
Composite arrhythmic endpoint (recurrence of ventricular tachyarrhythmias, sudden cardiac death), at 30 days	100	(21)	107	(23)	0.524	36	(15)	45	(19)	0.272
Composite arrhythmic endpoint (recurrence of ventricular tachyarrhythmias, sudden cardiac death), at 30 months	148	(27)	168	(30)	0.223	57	(24)	65	(27)	0.401
**Follow-up times**, *n* (%)										
Hospitalization total; days (median (IQR))	12 (7–20)		11 (5–22)		0.693	13 (8–23)		13 (7–24)		0.422
ICU time; days (median (IQR))	4 (1–8)		4 (2–10)		**0.001**	4 (1–9)		5 (2–10)		0.174
Follow-up; days (mean; median (range))	1183; 795(0–4655)		891; 263(0–4624)		**0.001**	1258; 1079 (0–4357)		1006; 624 (0–4626)		0.008

ICU, invasive care unit; IQR, interquartile range. * Mode of death was unknown in 14% of the patients at 30 days. Level of significance is *p* ≤ 0.05. Bold type indicates *p* ≤ 0.05.

**Table 4:** Uni- and multivariable Cox regression analysis with regard to 30-day all-cause mortality (primary endpoint).

	Univariable			Multivariable		
	HR	95% CI	*p* Value	HR	95% CI	*p* Value
Age	1.030	1.023–1.036	**0.001**	1.014	1.000–1.028	**0.042**
Males	0.843	0.718–0.990	**0.038**	1.232	0.837–1.814	0.291
Diabetes	1.213	1.031–1.428	**0.020**	0.948	0.672–1.336	0.759
Chronic Kidney disease	4.268	3.529–5.161	**0.001**	5.786	3.324–10.073	**0.001**
LVEF < 35%	1.322	1.070–1.633	**0.010**	1.260	1.090–1.458	**0.002**
Nonischemic cardiomyopathy	0.285	0.165–0.494	**0.001**	0.588	0.208–1.660	0.316
Coronary angiography	0.463	0.398–0.538	0.001	0.505	0.358–0.713	**0.001**
Electrophysiological examination	0.030	0.015–0.060	**0.001**	0.261	0.063–1.075	0.063
Presence of ICD	0.061	0.042–0.089	**0.001**	0.096	0.049–0.185	**0.001**
Hemoglobin	0.810	0.783–0.838	**0.001**	0.974	0.092–1.052	0.500
Serum potassium	1.481	1.354–1.621	**0.001**	1.130	0.954–1.339	0.157
high cTNI	2.004	1.603–2.505	0.001	1.541	1.088–2.182	**0.015**

CI, confidence interval; HR, hazard ratio; ICD, implantable cardioverter defibrillator; LVEF, left ventricular ejection faction. Level of significance is *p* < 0.05. Bold type indicates statistical significance.

**Table 5:** Uni- and multivariable hazard ratios for “high cTNI” with regard to 30-day all-cause mortality (primary endpoint) within prespecified subgroups.

	Univariable			Multivariable *		
	HR	95% CI	*p* Value	HR	95% CI	*p* Value
Ventricular tachycardia	2.694	1.762–4.121	**0.001**	2.333	1.276–4.267	**0.006**
Ventricular fibrillation	1.496	1.151–1.944	**0.003**	1.708	1.177–2.478	**0.005**
STEMI	1.258	0.563–2.813	0.576	5.047	0.657–38.607	0.120
NSTEMI	2.030	1.249–3.300	**0.004**	2.661	1.126–6.289	**0.026**
No myocardial infarction	2.364	1.772–3.152	**0.001**	1.963	1.331–2.897	**0.001**
Nonischemic cardiomyopathy	6.299	0.703–56.414	0.100	12.164	0.999–148.093	**0.050**
Idiopathic ventricular tachyarrhythmias	2.674	1.730–4.135	0.001	2.628	1.223–5.651	**0.013**
**Patients with coronary angiography**						
Coronary artery disease	1.940	1.353–2.783	**0.001**	1.799	1.093–2.960	**0.021**
No coronary artery disease	6.421	2.679–15.390	**0.001**	5.466	1.725–17.316	**0.004**
Multivessel disease	1.985	1.292–3.048	**0.002**	1.736	0.950–3.171	0.073
Presence of CABG	3.002	0.924–9.752	0.067	2.048	0.473–8.867	0.338
Chronic total occlusion	2.638	1.388–5.016	**0.003**	1.916	0.859–4.272	0.112
Ischemic cardiomyopathy	2.106	1.476–3.004	**0.001**	1.801	1.102–2.942	**0.019**

CI, confidence interval; HR, hazard ratio; CABG, coronary artery bypass grafting; LVEF, left ventricular ejection faction; NSTEMI, non-ST-segment elevation myocardial infarction. * Multivariable models were adjusted for age, sex, diabetes mellitus, chronic kidney disease, LVEF < 35% and nonischemic cardiomyopathies. Level of significance is *p* < 0.05. Bold type indicates statistical significance.

## Data Availability

The datasets used and/or analyzed during the current study are available from the corresponding author on reasonable request.

## Supplementary Materials

The following are available online at https://www.mdpi.com/article/10.3390/jcm11112987/s1, Table S1: Prognostic performance of cTNI for 30-day all-cause mortality in pre-specified subgroups, Figure S1: Prognostic impact of cTNI on 30-day all-cause mortality in patients with NICMP and idiopathic ventricular tachyarrhythmias.

## References

[1] Burrell A.J., Pellegrino V.A., Wolfe R., Wong W.K., Cooper D.J., Kaye D.M., Pilcher D.V. (2015). Long-term survival of adults with cardiogenic shock after venoarterial extracorporeal membrane oxygenation. J. Crit. Care.

[2] Wong C.X., Brown A., Lau D.H., Chugh S.S., Albert C.M., Kalman J.M., Sanders P. (2019). Epidemiology of Sudden Cardiac Death: Global and Regional Perspectives. Heart Lung Circ..

[3] Maggioni A.P., Anker S.D., Dahlström U., Filippatos G., Ponikowski P., Zannad F., Amir O., Chioncel O., Leiro M.C., Drozdz J. (2013). Are hospitalized or ambulatory patients with heart failure treated in accordance with European Society of Cardiology guidelines? Evidence from 12,440 patients of the ESC Heart Failure Long-Term Registry. Eur. J. Heart Fail..

[4] Myerburg R.J., Junttila M.J. (2012). Sudden cardiac death caused by coronary heart disease. Circulation.

[5] Koplan B.A., Stevenson W.G. (2009). Ventricular tachycardia and sudden cardiac death. Mayo Clin. Proc..

[6] Everett B.M., Moorthy M.V., Tikkanen J.T., Cook N.R., Albert C.M. (2020). Markers of Myocardial Stress, Myocardial Injury, and Subclinical Inflammation and the Risk of Sudden Death. Circulation.

[7] Priori S.G., Blomström-Lundqvist C., Mazzanti A., Blom N., Borggrefe M., Camm J., Elliott P.M., Fitzsimons D., Hatala R., Hindricks G. (2015). 2015 ESC Guidelines for the management of patients with ventricular arrhythmias and the prevention of sudden cardiac death: The Task Force for the Management of Patients with Ventricular Arrhythmias and the Prevention of Sudden Cardiac Death of the European Society of Cardiology (ESC)Endorsed by: Association for European Paediatric and Congenital Cardiology (AEPC). Eur. Heart J..

[8] Hussein A.A., Gottdiener J.S., Bartz T.M., Sotoodehnia N., deFilippi C., Dickfeld T., Deo R., Siscovick D., Stein P.K., Lloyd-Jones D. (2013). Cardiomyocyte injury assessed by a highly sensitive troponin assay and sudden cardiac death in the community: The Cardiovascular Health Study. J. Am. Coll. Cardiol..

[9] Patton K.K., Sotoodehnia N., DeFilippi C., Siscovick D.S., Gottdiener J.S., Kronmal R.A. (2011). N-terminal pro-B-type natriuretic peptide is associated with sudden cardiac death risk: The Cardiovascular Health Study. Heart Rhythm.

[10] Korngold E.C., Januzzi J.L., Gantzer M.L., Moorthy M.V., Cook N.R., Albert C.M. (2009). Amino-terminal pro-B-type natriuretic peptide and high-sensitivity C-reactive protein as predictors of sudden cardiac death among women. Circulation.

[11] Goldberger J.J., Bonow R.O., Cuffe M., Dyer A., Rosenberg Y., O’Rourke R., Shah P.K., Smith S.C. (2010). beta-Blocker use following myocardial infarction: Low prevalence of evidence-based dosing. Am. Heart J..

[12] Shah N.N., Ayyadurai P., Saad M., Kosmas C.E., Dogar M.U., Patel U., Vittorio T.J. (2020). Galactin-3 and soluble ST2 as complementary tools to cardiac MRI for sudden cardiac death risk stratification in heart failure: A review. JRSM Cardiovasc. Dis..

[13] Sutanto H., Lyon A., Lumens J., Schotten U., Dobrev D., Heijman J. (2020). Cardiomyocyte calcium handling in health and disease: Insights from in vitro and in silico studies. Prog. Biophys. Mol. Biol..

[14] Katrukha I.A. (2013). Human cardiac troponin complex. Structure and functions. Biochemistry.

[15] Chaulin A.M. (2021). Cardiac Troponins Metabolism: From Biochemical Mechanisms to Clinical Practice (Literature Review). Int. J. Mol. Sci..

[16] Shomanova Z., Ohnewein B., Schernthaner C., Höfer K., Pogoda C.A., Frommeyer G., Wernly B., Brandt M.C., Dieplinger A.M., Reinecke H. (2020). Classic and Novel Biomarkers as Potential Predictors of Ventricular Arrhythmias and Sudden Cardiac Death. J. Clin. Med..

[17] Ghersin I., Zahran M., Azzam Z.S., Suleiman M., Bahouth F. (2020). Prognostic value of cardiac troponin levels in patients presenting with supraventricular tachycardias. J. Electrocardiol..

[18] Nakamura H., Niwano S., Fukaya H., Murakami M., Kishihara J., Satoh A., Yoshizawa T., Oikawa J., Ishizue N., Igarashi T. (2017). Cardiac troponin T as a predictor of cardiac death in patients with left ventricular dysfunction. J. Arrhythm.

[19] Schupp T., Behnes M., Weiß C., Nienaber C., Lang S., Reiser L., Bollow A., Taton G., Reichelt T., Ellguth D. (2018). Beta-Blockers and ACE Inhibitors Are Associated with Improved Survival Secondary to Ventricular Tachyarrhythmia. Cardiovasc. Drugs Ther..

[20] Apple F.S., Collinson P.O. (2012). Analytical characteristics of high-sensitivity cardiac troponin assays. Clin. Chem..

[21] Ibanez B., James S., Agewall S., Antunes M.J., Bucciarelli-Ducci C., Bueno H., Caforio A.L.P., Crea F., Goudevenos J.A., Halvorsen S. (2018). 2017 ESC Guidelines for the management of acute myocardial infarction in patients presenting with ST-segment elevation: The Task Force for the management of acute myocardial infarction in patients presenting with ST-segment elevation of the European Society of Cardiology (ESC). Eur. Heart J..

[22] Behnes M., Mashayekhi K., Weiß C., Nienaber C., Lang S., Reiser L., Bollow A., Taton G., Reichelt T., Ellguth D. (2018). Prognostic Impact of Acute Myocardial Infarction in Patients Presenting With Ventricular Tachyarrhythmias and Aborted Cardiac Arrest. J. Am. Heart Assoc..

[23] Elliott P., Andersson B., Arbustini E., Bilinska Z., Cecchi F., Charron P., Dubourg O., Kühl U., Maisch B., McKenna W.J. (2008). Classification of the cardiomyopathies: A position statement from the European Society Of Cardiology Working Group on Myocardial and Pericardial Diseases. Eur. Heart J..

[24] Elliott P.M. (2013). Classification of cardiomyopathies: Evolution or revolution?. J. Am. Coll. Cardiol..

[25] Rapezzi C., Arbustini E., Caforio A.L., Charron P., Gimeno-Blanes J., Heliö T., Linhart A., Mogensen J., Pinto Y., Ristic A. (2013). Diagnostic work-up in cardiomyopathies: Bridging the gap between clinical phenotypes and final diagnosis. A position statement from the ESC Working Group on Myocardial and Pericardial Diseases. Eur. Heart J..

[26] Rusnak J., Behnes M., Weiß C., Nienaber C., Reiser L., Schupp T., Bollow A., Taton G., Reichelt T., Ellguth D. (2020). Non-ischemic compared to ischemic cardiomyopathy is associated with increasing recurrent ventricular tachyarrhythmias and ICD-related therapies. J. Electrocardiol..

[27] Behnes M., Akin I., Kuche P., Schupp T., Reiser L., Bollow A., Taton G., Reichelt T., Ellguth D., Engelke N. (2020). Coronary chronic total occlusions and mortality in patients with ventricular tachyarrhythmias. EuroIntervention.

[28] Behnes M., Mashayekhi K., Kuche P., Kim S.H., Schupp T., von Zworowsky M., Reiser L., Bollow A., Taton G., Reichelt T. (2021). Prognostic impact of coronary chronic total occlusion on recurrences of ventricular tachyarrhythmias and ICD therapies. Clin. Res. Cardiol..

[29] Yin J., Samawi H., Linder D. (2016). Improved nonparametric estimation of the optimal diagnostic cut-off point associated with the Youden index under different sampling schemes. Biom. J..

[30] Yin J., Tian L. (2014). Joint confidence region estimation for area under ROC curve and Youden index. Stat. Med..

[31] Osman J., Tan S.C., Lee P.Y., Low T.Y., Jamal R. (2019). Sudden Cardiac Death (SCD)—Risk stratification and prediction with molecular biomarkers. J. Biomed. Sci..

[32] Pearson D.A., Wares C.M., Mayer K.A., Runyon M.S., Studnek J.R., Ward S.L., Kraft K.M., Heffner A.C. (2015). Troponin Marker for Acute Coronary Occlusion and Patient Outcome Following Cardiac Arrest. West J. Emerg. Med..

[33] Gilje P., Koul S., Thomsen J.H., Devaux Y., Friberg H., Kuiper M., Horn J., Nielsen N., Pellis T., Stammet P. (2016). High-sensitivity troponin-T as a prognostic marker after out-of-hospital cardiac arrest—A targeted temperature management (TTM) trial substudy. Resuscitation.

[34] Lopez E.M., Malhotra R. (2019). Ventricular Tachycardia in Structural Heart Disease. J. Innov. Card. Rhythm Manag..

[35] Ajijola O.A., Tung R., Shivkumar K. (2014). Ventricular tachycardia in ischemic heart disease substrates. Indian Heart J..

[36] Liu L., Liu S., Shen L., Tu B., Hu Z., Hu F., Zheng L., Ding L., Fan X., Yao Y. (2020). Correlations between cardiac troponin I and nonsustained ventricular tachycardia in hypertrophic obstructive cardiomyopathy. Clin. Cardiol..

