# Supplementary material for: Prognostic Value of Cardiac Troponin I in Patients with Ventricular Tachyarrhythmias
Source: J Clin Med. 2022 May 25;11(11):2987. doi: 10.3390/jcm11112987 (PMC9181556; doi:10.3390/jcm11112987)
Supplement: Supplementary file 1 [file jcm-11-02987-s001.zip › jcm-1695479-supplementary.pdf]

Supplemental Table S1. Prognostic performance of cTNI for 30-day all-cause mortality in pre-specified subgroups.

Subgroup	AUC	95% CI	p value
Coronary artery disease	0.684	0.598-0.769	**0.044**
STEMI	0.559	0.429-0.690	0.379
NSTEMI	0.627	0.530-0.724	**0.050**
Non-AMI	0.698	0.637-0.760	**0.001**
Idiopathic ventricular tachyarrhythmias	0.726	0.632-0.820	**0.001**
Coronary angiography	0.637	0.575-0.700	**0.032**
Multi-vessel disease	0.616	0.524-0.707	**0.047**
CABG	0.636	0.314-0.928	0.314
CTO	0.637	0.492-0.783	0.075

AMI, acute myocardial infarction; AUC, area under the curve; CABG, coronary artery bypass grafting; CTO, chronic total occlusion; (N)STEMI, (non-) ST-segment elevation myocardial infarction.

Level of significance p<0.05.

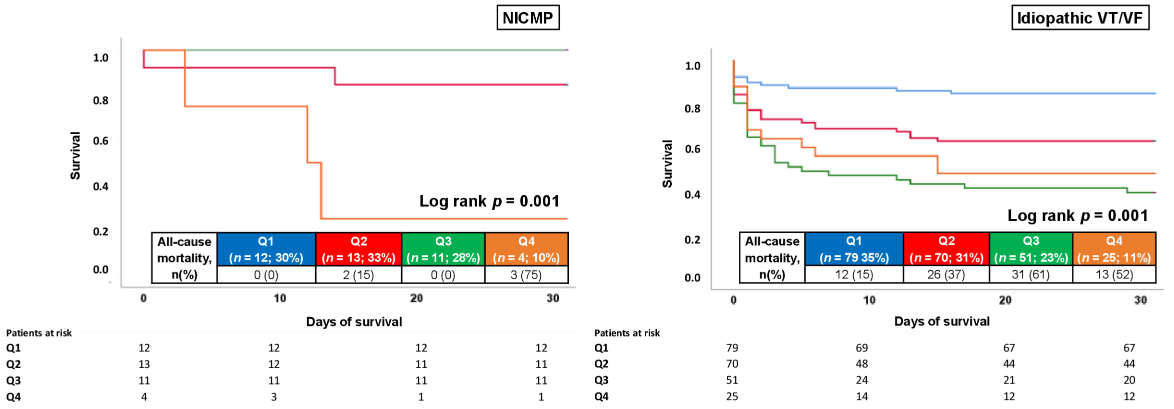

Figure S1. Prognostic impact of cTNI on 30-day all-cause mortality in patients with NICMP and idiopathic ventricular tachyarrhythmias.
